# Early versus delay oral feeding for patients after upper gastrointestinal surgery: a systematic review and meta-analysis of randomized controlled trials

**DOI:** 10.1186/s12935-022-02586-y

**Published:** 2022-04-29

**Authors:** Huachu Deng, Baibei Li, Xingan Qin

**Affiliations:** 1 Department of Gastrointestinal and Gland Surgery, The First Affiliated Hospital of Guangxi Medical University, Nanning, Guangxi China; 2 Department of Hepatobiliary, The First Affiliated Hospital of Guangxi Medical University, Nanning, Guangxi China

**Keywords:** Early oral feeding, Upper gastrointestinal surgery, Meta-analysis

## Abstract

**Purpose:**

To evaluate the efficacy and safety of early oral feeding (EOF) in patients after upper gastrointestinal surgery through meta-analysis of randomized controlled trials (RCTs).

**Methods:**

We analyzed the endpoints of patients including the length of stay (LOS), time of first exhaust, anastomotic leakage and pneumonia from included studies. And we retrieved RCTs from medical literature databases. Weighted mean difference (WMD), risk ratios (RR) and 95% confidence intervals (CI) were calculated to compare the endpoints.

**Results:**

In total, we retrieved 12 articles (13 trial comparisons) which contained 1771 patients. 887 patients (50.1%) were randomized to EOF group whereas 884 patients (49.9%) were randomized to delay oral feeding group. The result showed that compared with the delay oral feeding group, EOF after upper gastrointestinal surgery significantly shorten the LOS [WMD = − 1.30, 95% CI − 1.79 to − 0.80, I^2^ = 0.0%] and time of first exhaust [WMD = − 0.39, 95% CI − 0.58 to − 0.20, I^2^ = 62.1%]. EOF also reduced the risk of pneumonia (RR: 0.74, 95% CI 0.55 to 0.99, I^2^ = 0.0%). There is no significant difference in the risk of anastomotic leak, anastomotic bleeding, abdominal abscess, reoperation, readmission and mortality.

**Conclusions:**

Overall, compared with the traditional oral feeding, EOF could shorten the LOS and time of first exhaust without increasing complications after upper gastrointestinal surgery.

## Introduction

Upper gastrointestinal surgery mainly refers to the operation of esophagus, stomach, duodenum, liver or pancreas [1]. Its common indication is cancer. Anastomotic leakage after upper gastrointestinal surgery is a major source of morbidity as it can lead to severe infection and increase the risk of fatal sequelae in the absence of reasonable treatment [2]. After upper gastrointestinal surgery, patients often have difficulty in eating, increased catabolism, weakened anabolism and decreased immune function, which will result in (or aggravating) malnutrition [3]. It may increase the incidence of postoperative complications and mortality.

As for the timing of eating after upper gastrointestinal surgery, most surgeons still follow the traditional principle of restoring intestinal function after anal exhaust and then eating gradually. This approach is based on avoiding complications that may be caused by excessive gastrointestinal volume or early gastrointestinal stimulation, including nausea, vomiting, aspiration pneumonia, anastomotic leakage and so on [4]. However, fasting from postoperative to anal exhaust will lead to insufficient enteral nutrition and inhibit the secretion of saliva and digestive glands [5]. It will also delay the recovery of digestive system function and increase the risk of potential pathogen infection and microbial translocation which seriously affect postoperative recovery and wound healing [6]. After all, nutritional status is an important factor affecting postoperative recovery.

Recently, enhanced recovery after surgery (ERAS) has attracted more and more attention, which requires multidisciplinary teamwork to accelerate recovery during perioperative care [7]. Early oral feeding (EOF) is one of the most important elements of ERAS [8]. A considerable number of literatures have confirmed that EOF can effectively reduce the length of stay and accelerate the recovery of gastrointestinal function without increasing postoperative complications [9–12]. In 2016, Willcutts et al. [13] made a meta-analysis to compare the effect of EOF on clinical outcome after upper gastrointestinal surgery. They concluded that compared with conventional feeding, postoperative EOF was associated with a shorter length of hospital stay and is not associated with an increase in clinically relevant complications. In the past 5 years, more and more randomized controlled trials (RCTs) about the effect of EOF in upper gastrointestinal surgery have been continuously published. However, the clinical application of EOF is still controversial, and it is not widely used. It remains to be seen whether the conclusion of Willcutts’s study is still applicable. It is necessary to make an updated meta-analysis.

## Methods

### Search strategy

Two investigators searched published articles according to the Preferred Reporting Items for Systematic Reviews and Meta-Analyses (PRISMA) to compare the efficacy and safety of EOF in patients after upper gastrointestinal surgery [14]. We conducted a systematic search for RCTs in databases such as the Cochrane Library, Embase, Baidu Schilar, PubMed, and Google Scholar with language restrictions to English and publication dates restricted to April 14, 2021. The following keywords and MeSH terms were used to search: (“early oral feeding” or “EOF” or “enhanced recovery after surgery” or “ERAS” or “direct oral feeding”) and (“upper gastrointestinal surgery” or “esophagectomy” or “gastrectomy” or “anastomosis” or “gastrointestinal” or “surgery”). The retrieved studies were screened by one author and double-reviewed by another author. When there was a dispute, a third author was involved in the discussion and made a decision together. All data was extracted from published studies, therefore patient consent and ethical approval were not required.

### Exclusion and inclusion criteria

Exclusion criteria: (1) Semi-randomized or non-randomized trials; (2) Animal experiments; (3) Nonclinical trials or case reports; and (4) Articles with incorrect or incomplete data or articles whose data could not be extracted.

Inclusion criteria: (1) Studies that compared EOF and delay oral feeding for patients after upper gastrointestinal surgery; (2) The study was a RCT; (3) Baseline characteristics (e.g., age, gender and type of surgery) were not statistically different between two groups; (4) The study subjects were patients undergoing upper gastrointestinal surgery; (5) one of groups was applied EOF; (6) The language of the studies was restricted to English; and (7) Included studies provided sufficient data for the analysis.

### Endpoints

The primary effective endpoints were length of stay (LOS) and time of first exhaust. The safety endpoints were anastomotic leak, anastomotic bleeding, abdominal abscess, reoperation, readmission, mortality and pneumonia.

### Data extraction

The content of the included studies was independently reviewed by two authors. Two authors extracted the primary endpoints and a third author verified endpoints. The following main information was extracted from the included studies: year of publication, first author's name, time period, country of patients, population, mean age, operation type, endpoints in each study and intervention. If the included studies required clarification, we contacted the first author of the included study. When there was a disagreement, we resolved it by consensus or consultation with a third author.

### Assessment of risk of bias

Two authors independently evaluated the quality of the methodology according to the Cochrane Risk of Bias criteria [15]. Each quality items were classified as high risk, low risk, and some concerns. There are 5 items were used to estimate bias for each included studies, including bias due to deviations from intended interventions, bias arising from the randomization process, bias in measurement of the outcome, bias due to missing outcome data, bias in selection of the reported result.

### Statistical analysis

We used Stata (version 12.0) to analyze and pool the included studies results. We recorded pooled results by weignted mean difference (WMD), risk ratios (RR), and 95% confidence intervals (CI) with two-sided P-values. There were considered to be statistically significant when P-values < 0.05. I^2^ test was used to evaluate heterogeneity. The heterogeneity was considered to be substantial and the random effect model was used when I^2^ > 50%, while the fixed effect model was used when I^2^ < 50%. If there were more than ten studies assessed one endpoint, we examined the publication bias and explored sources of heterogeneity by funnel plot [16, 17].

## Results

### Features of the studies included and retrieved data

**Fig. 1:**
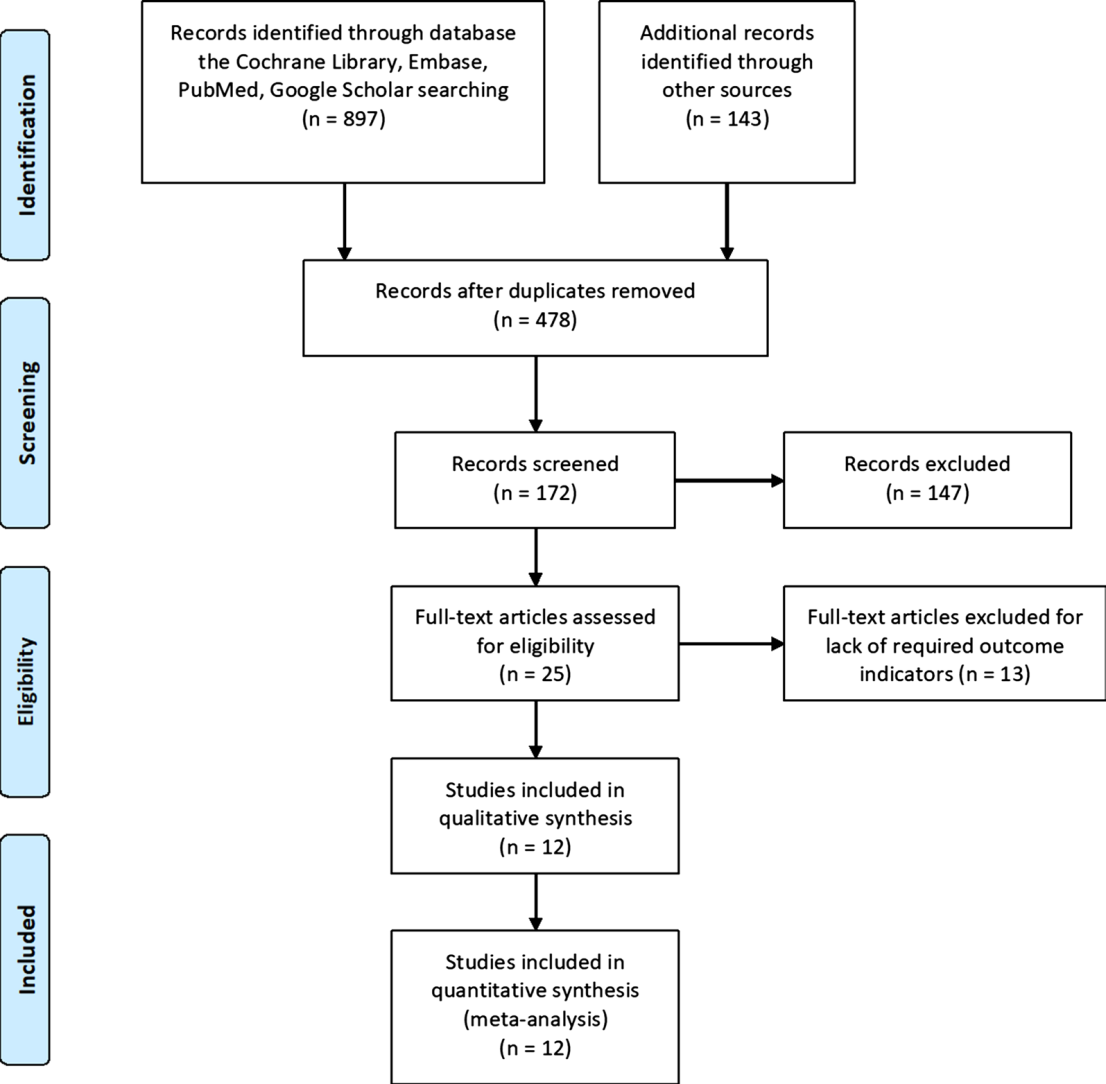
Flow diagram of included studies selection

According to PRISMA guidelines, 478 studies were enrolled. We then eliminated a portion of the articles by screening the abstracts, and identified the final articles for inclusion by reading the full text. Finally, 12 studies [9, 18–28] (13 trial comparisons) were included which contained 1771 patients as shown in Fig. 1. 887 patients (50.1%) were randomized to EOF group whereas 884 patients (49.9%) were randomized to delay oral feeding group. All included studies were RCTs. The basic characteristics of the included studies were described in Table 1.

**Table 1:** Characteristics of studies included in meta-analysis

Authors	Year	Time period	Country	Sample size		Average age (years) mean ± sd		Operation type	Intervention		Endpoints
				EOF	Control	EOF	Control		EOF	Control	
Suresh et al	2000	1999–2000	India	17	16	48.9	55.7	Cervical esophagogastrostomy	NGT removed POD3. Surgical J tube. Oral feeding POD3, if no leak, started with liquids then to semisolids the same day. If leak, started on TF	NGT removed POD3. Surgical J tube. Oral feeding POD5, if no leak, started with liquids then to semisolids the same day. If leak, started on TF	Anastomotic leaks
Hirao et al	2005	1999–2002	Japan	53	50	61.6 ± 10.4	61.0 ± 13.3	Open distal gastrectomy—Billroth I or Roux-en-Y—GD or GJ	NGT removed POD1. Wateron POD1, clears POD2, solid diet on POD3	NGT removed POD1. 200 mL water POD4, clear liquids POD6, and solid diet POD10	LOS, anastomotic leaks, abdominal abscess, reoperation, mortality, pneumonia
Lassen et al	2008	2001–2006	Norway	220	227	63.0 ± 14.4	65.0 ± 13.3	Hepatic, pancreatic, esophageal, gastric resections, bilioenteric and gastroenteric bypass, and others where traditionally pts are NPO after surgery	NGT removed at least by POD1. Allowed food at will POD1. Pts were instructed to begin intake carefully and to “adjust according to tolerance”	NGT removed at least by POD1. Oral diet POD6. Feeding jejunostomy. Saline at 20 mL/h until POD1 morning. Then TF started at 20 mL/h. Increased by 20 mL/h/d, as tolerated, to goal of 80 mL/h	Anastomotic leaks, abdominal abscess, reoperation, readmission, mortality, pneumonia
Hur et al	2011	2008–2009	Korea	28	26	NA	NA	Open partial or total gastrectomy; Roux-en-Y, Billroth I and Billroth II	Sips of water on POD1, soft diet POD3; no routine NGT	Sips of water POD3 and soft diet POD6, as tolerated; no routine NGT	LOS, times to first exhaust, anastomotic leaks, anastomotic bleeding, reoperation, readmission
Mi et al	2012	2010–2011	China	30	30	57.2 ± 9.5	60.0 ± 10.3	Proximal subtotal gastrectomy, distal subtotal gastrectomy, total gastrectomy	POD1-water. 500 mL Jevity 1 Cal by mouth and water on POD2. Full liquid diet with 1000 mL Jevity Cal on POD3. Then increased to semi liquid diet	Feeding nasal-intestinal tube. Removed tube after flatus and started water p.o. Increased water, liquid, or semiliquid diet gradually until discharge	LOS, times to first exhaust, anastomotic leaks, mortality, pneumonia
Peng et al	2014	2012–2012	China	42	36	51.1 ± 15.1	50.5 ± 13.6	Bilioenteric anastomosis	Water 5–6 h postoperation. Small amount of liquid diet on POD1. Transitioned to semi liquid diet/regular diet depending on patient’s tolerance. Used PN to supplement. NGT not reported	Feeding nasal-gastric tube placed. Fasting for solids and liquids, relying on TPN. Removed tube after flatus and started liquid diet, gradually transitioned to regular diet, supplemented with PN	LOS, times to first exhaust, time to first defecation, anastomotic leaks, abdominal abscess, pneumonia
Mahmoodzadeh et al	2015	2011–2012	Iran	54	55	64.2 ± 8.2	66.4 ± 7.7	Transthoracic esophagectomy (open). Total gastrectomies-Roux-en-Y EJ. Partial gastrectomies—Billroth I or II or Roux-en-Y GJ	NGT removed POD1. POD1-100 mL tea with sugar and gradually increased to 250 mL. If no nausea or emesis and flatus and bowel sounds, diet advanced to soft (500 mL cold soup every 8 h)	NPO, and NGT if needed, until bowel sounds returned and “resolution of ileus”	Anastomotic leaks, abdominal abscess, readmission, pneumonia
Sun et al	2018	2014–2015	China	140	140	63.0	63.0	Thoracolaparoscopic esophagectomy, complete truncal vagotomy	POD 1—liquid foods. POD 2—semi-liquid food and soft solid Foods. POD 4—stopped PN	POD1—nasogastric and nasoenteral feeding tubes. POD 7- removed nasogastric tube, allowed the same food as in the EOF group	Anastomotic leaks, anastomotic bleeding, readmission, mortality, pneumonia
Shimizu et al. a	2018	2014–2015	Japan	70	84	64.5	64.0	Distal gastrectomy	POD1-3—iEAT^®^ (a commercially available food, was used as the standardized diet for early oral feeding). POD4 and thereafter—ordinary hospital diets	Conventional nutritional management	Anastomotic leaks, anastomotic bleeding, abdominal abscess, readmission, pneumonia
Shimizu et al. b	2018	2014–2015	Japan	32	30	68.5	68.5	Total gastrectomy	POD1-3—iEAT^®^ (a commercially available food, was used as the standardized diet for early oral feeding). POD4 and thereafter—ordinary hospital diets	Conventional nutritional management	Anastomotic leaks, anastomotic bleeding, abdominal abscess, readmission, pneumonia
Gao et al	2019	2015–2017	China	101	97	56.3 ± 10.2	53.9 ± 11.6	Laparoscopic radical gastrectomy	POD2—oral fluid diet. POD3—Semi-liquid food and soft food. Insufficient intake of oral nutrition was supplemented by intravenous fluids	Patients were indwelled with nasogastric tube 30 min before surgery until the recovery of gastrointestinal function. Nasogastric tube extubation was performed until exhaust occurrence and a small amount of white gastric fluid was found in the tube	Times to first exhaust, time to first defecation, anastomotic leaks, anastomotic bleeding
Berkelmans et al	2020	2015–2018	Netherlands/Sweden	65	67	65	65	Minimally invasive esophagectomy	POD0—drink sips of water up to 250 cc. POD1—500 cc liquid oral Intake. POD5—gradually increased up to 1500 cc on. POD15—solid foods without restrictions	Patients were only allowed to drink clear liquids up to 250 cc/day. They received tube feeding via the jejunostomy and started oral intake on POD5, expanding this diet exactly the same as in the oral group	
Masood et al	2021	2018–2019	USA	16	18	52.59 ± 20.49	49.68 ± 17.51	Perforated duodenal ulcers undergoing emergency repair using Graham's patch repair or a modified Graham's patch repair	The NG tube and Foley catheter were removed within 12 h, and patients were allowed oral sips on day one with a gradual shift to liquid diet after 12 h; semisolid food was started after 24 h	The Foley catheter and NG tube remained for 48 h following surgery, and patients remained nil per os (NPO; i.e., nothing by mouth) for three days and started with oral sips after 72 h	Anastomotic leaks, pneumonia

*NGT* Nasogastric tube, *EJ* esophagojejunostomy, *GD* gastroduodenostomy, *GJ* gastrojejunostomy, *PD* pancreaticoduodenectomy, *TF* tube feeding, *LOS* length of stay, *PN* parenteral nutrition, *NPO* nil per os (i.e., nothing by mouth), *POD* postoperative day, *NG * Nasogastric

### Assessment of quality of the studies

Two authors evaluated the quality of the retrieved studies by The Cochrane Risk of Bias criteria [15]. 12 studies [9, 18–28] described random sequence generation and allocation concealment. None of the studies described other biases. The included studies were all RCTs. The literature quality score was shown in Table 2.

**Table 2:** Assessment of methodological quality of included studies

Study	Bias arising from the randomisation process	Bias due to deviations from intended interventions	Bias due to missing outcome data	Bias in measurement of the outcome	Bias in selection of the reported result	Overall bias
Suresh et al.	Low	Low	Low	Low	Low	Low
Hirao et al.	Some concerns	Low	Low	Low	Low	Some concerns
Lassen et al.	Low	Low	Low	Low	Low	Low
Hur et al.	Low	Low	Low	Low	Low	Low
Mi et al.	Low	Low	Low	Low	Low	Low
Peng et al.	Low	Low	Low	Low	Low	Low
Mahmoodzadeh et al.	Low	Low	Low	Low	Low	Low
Sun et al.	Low	Low	Low	Low	Low	Low
Shimizu et al.	Low	Low	Low	Low	Low	Low
Gao et al.	Low	Low	Low	Low	Low	Low
Berkelmans et al.	Low	Low	Low	Low	Low	Low
Masood et al.	Low	Low	Low	Low	Low	Low

### Endpoints

#### Length of stay (LOS)

**Fig. 2:**
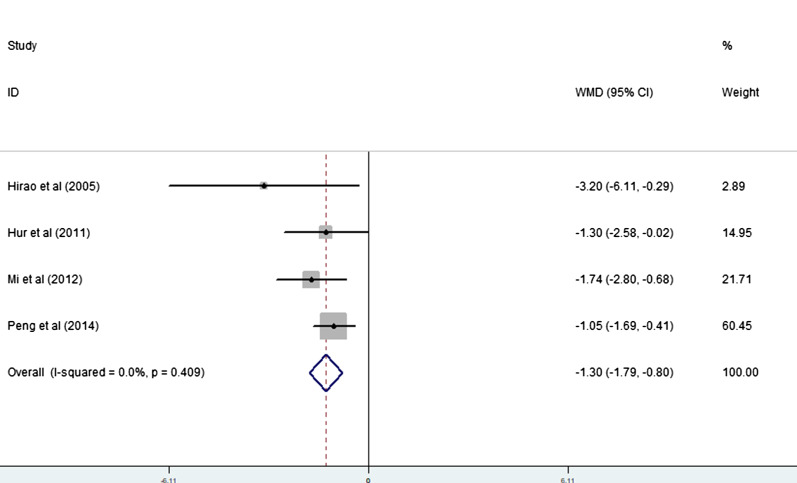
Forest plot of the LOS in EOF group and the DOF group. *WMD* weighted mean difference, *LOS* length of stay, *EOF* early oral feeding, *DOF* delay oral feeding

Four studies [19–22] (4 trial comparisons) reported LOS. Compared to the control group, the LOS in the EOF group was significantly shorter with statistical differences [WMD = − 1.30, 95% CI − 1.79 to − 0.80, I^2^ = 0.0%] as showed in Fig. 2. The fixed effect model was applied.

#### Time of first exhaust

**Fig. 3:**
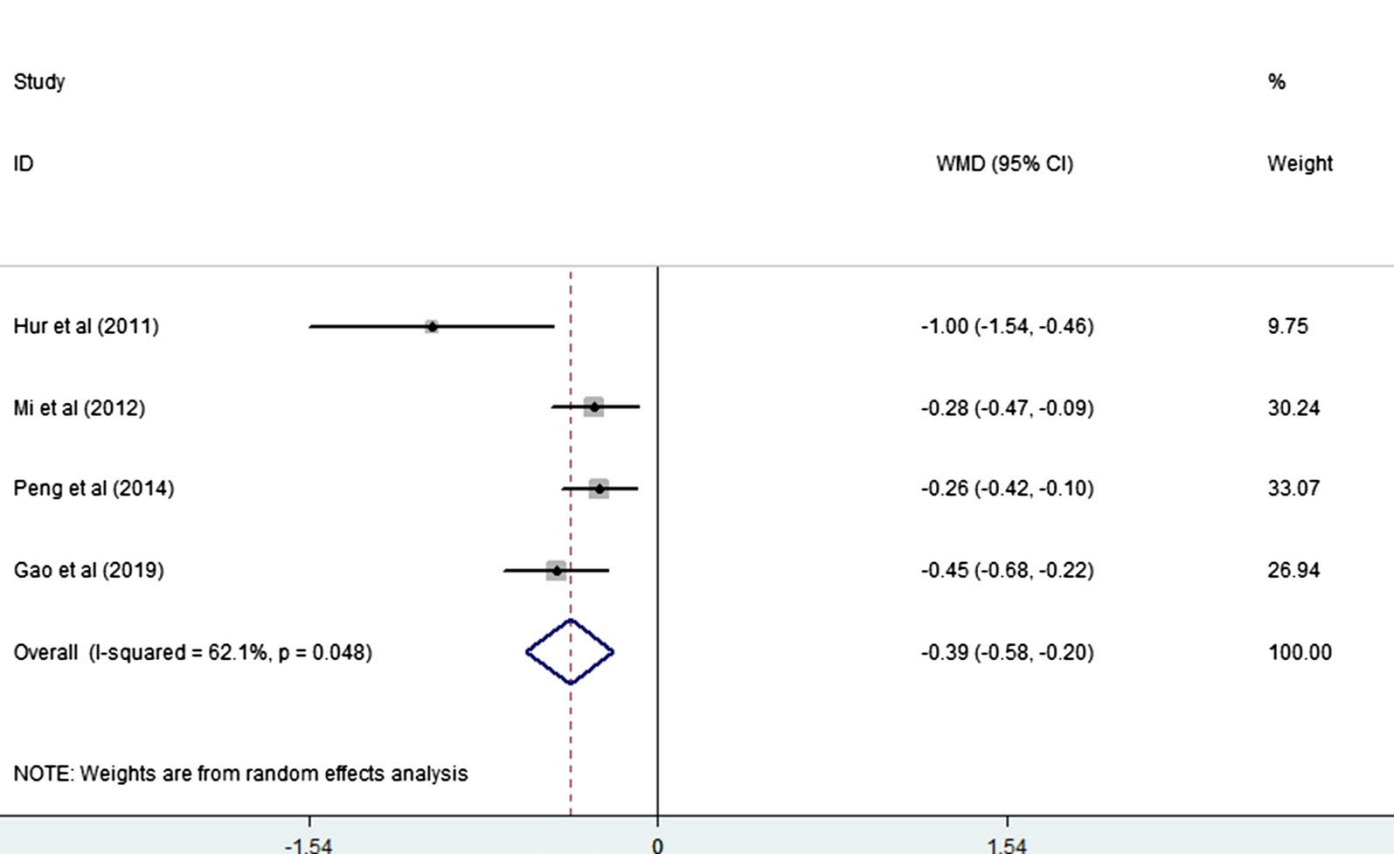
Forest plot of the time of first exhaust in EOF group and the DOF group. *WMD* weighted mean difference, *EOF* early oral feeding, *DOF* delay oral feeding

**Fig. 4:**
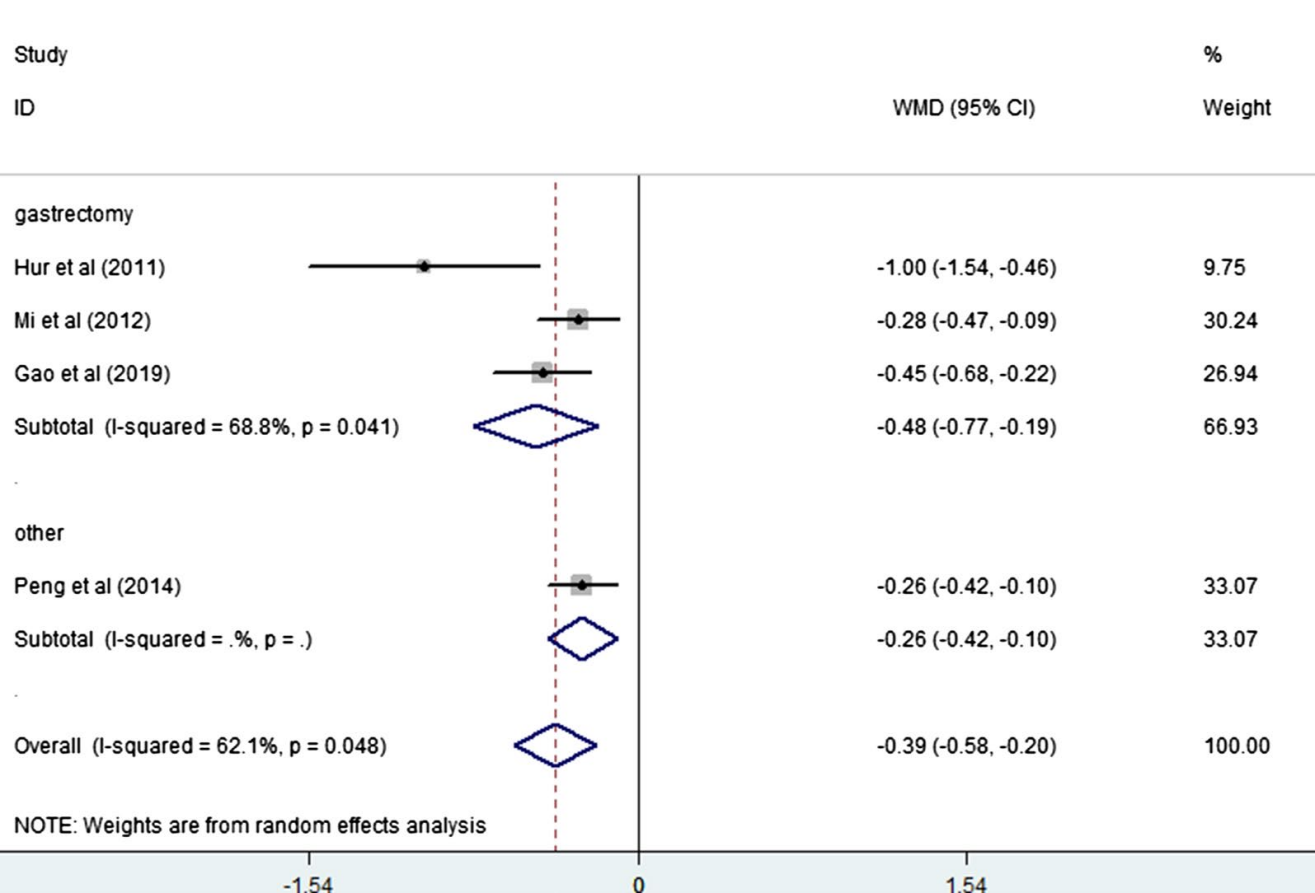
Subgroup analysis of time of first exhaust in EOF group and the DOF group. *WMD* weighted mean difference, *EOF* early oral feeding, *DOF* delay oral feeding

Four studies [20–22, 26] (4 trial comparisons) reported time of first exhaust. Compared to the control group, time of first exhaust in the EOF group was significantly shorter with statistical differences [WMD = − 0.39, 95% CI − 0.58 to − 0.20, I^2^ = 62.1%] as showed in Fig. 3. The random effect model was applied. And we performed a subgroup analysis by type of surgery. The results of the subsequent subgroup analysis showed that compared to patients undergoing other type of surgery (such as bilioenteric anastomosis) [RR = − 0.26, 95% CI − 0.42 to − 0.10], the effect in those patients undergoing gastrectomy was more significant [RR = − 0.48, 95% CI − 0.77 to − 0.19] as shown in Fig. 4.

#### Safety endpoint

**Fig. 5:**
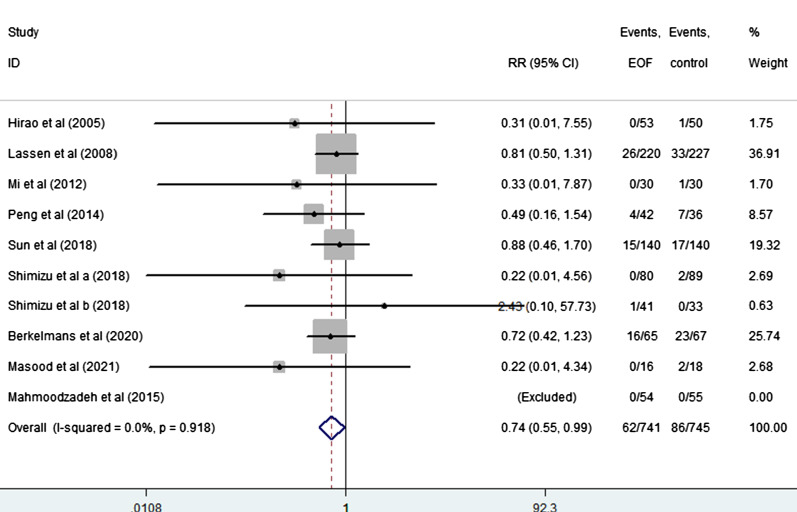
Forest plot of the risk of pneumonia in EOF group and DOF group. *RR* risk ratio, *EOF* early oral feeding, *DOF* delay oral feeding

**Fig. 6:**
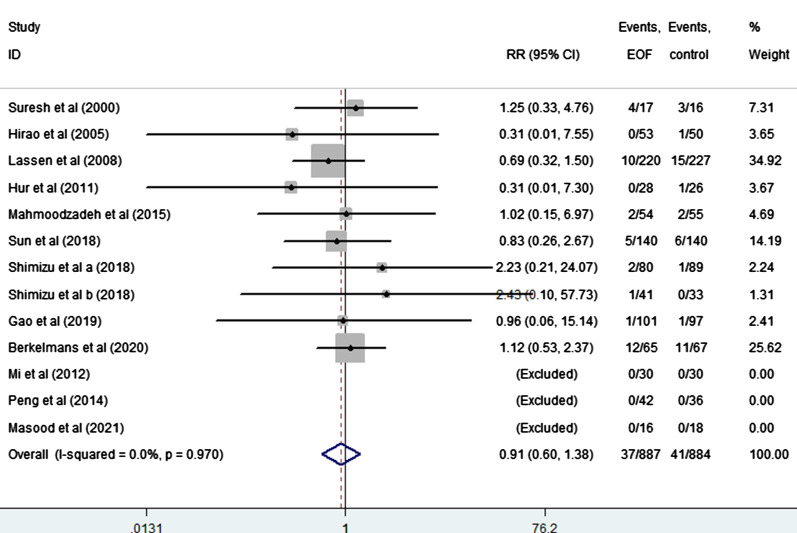
Forest plot of the risk of anastomotic leak in EOF group and DOF group. *RR* risk ratio, *EOF* early oral feeding, *DOF* delay oral feeding

**Fig. 7:**
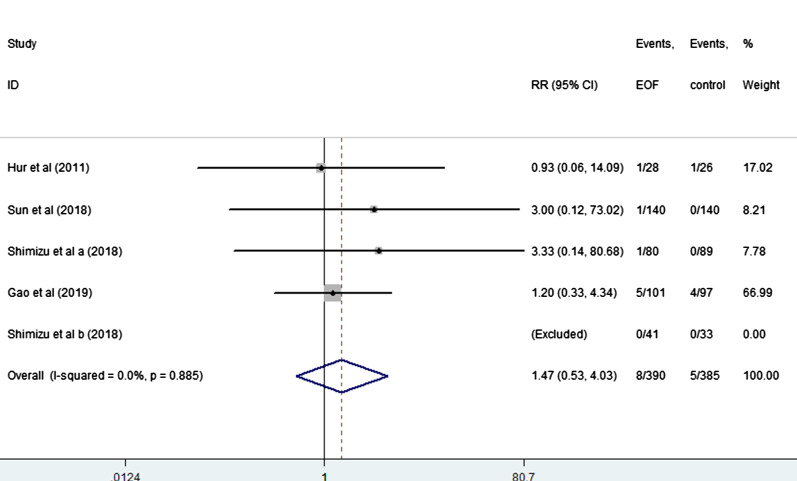
Forest plot of the risk of anastomotic bleeding in EOF group and the DOF group. *RR* risk ratio, *EOF* early oral feeding, *DOF* delay oral feeding

**Fig. 8:**
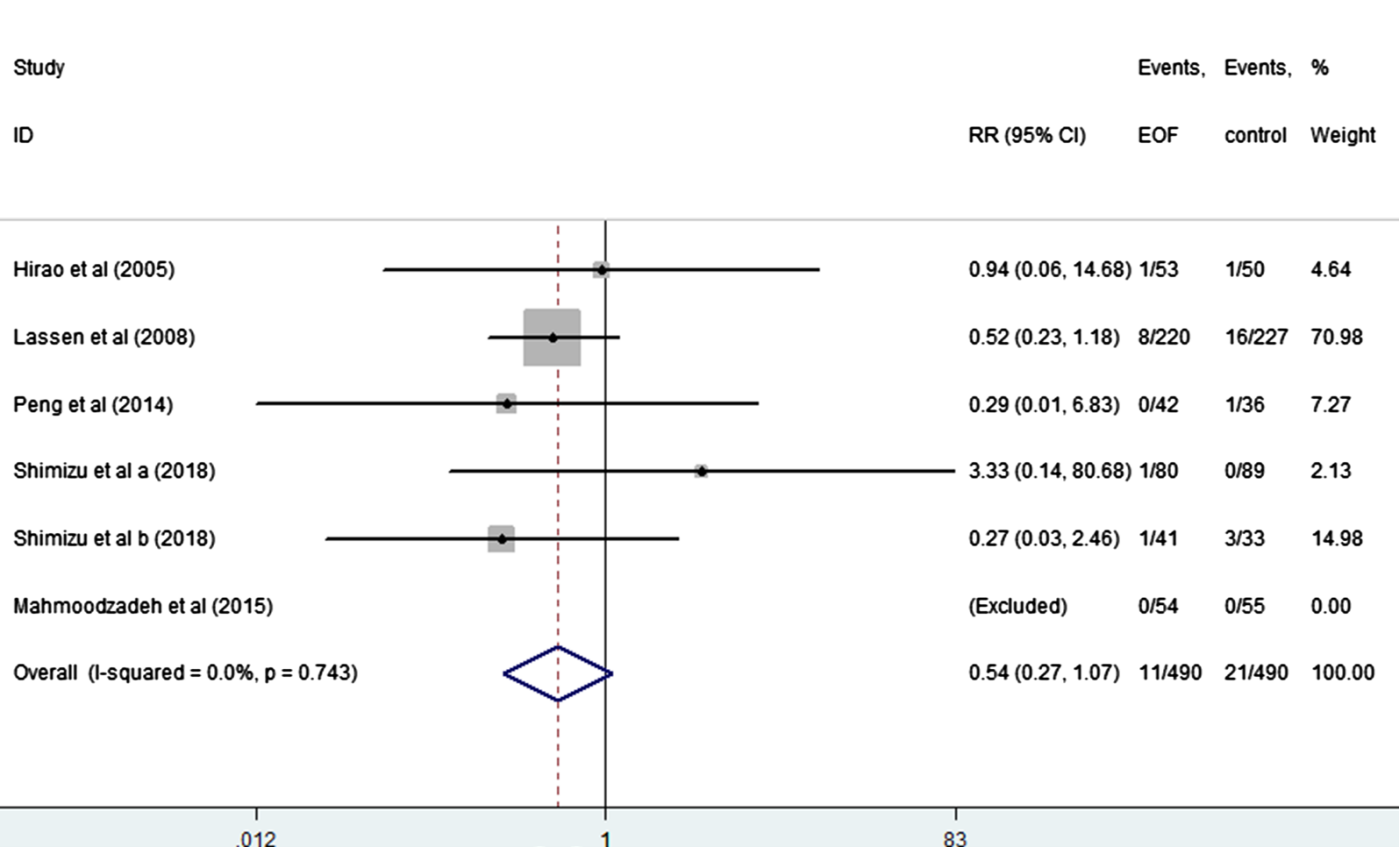
Forest plot of the risk of abdominal abscess in EOF group and the DOF group. *RR* risk ratio, *EOF* early oral feeding, *DOF* delay oral feeding

**Fig. 9:**
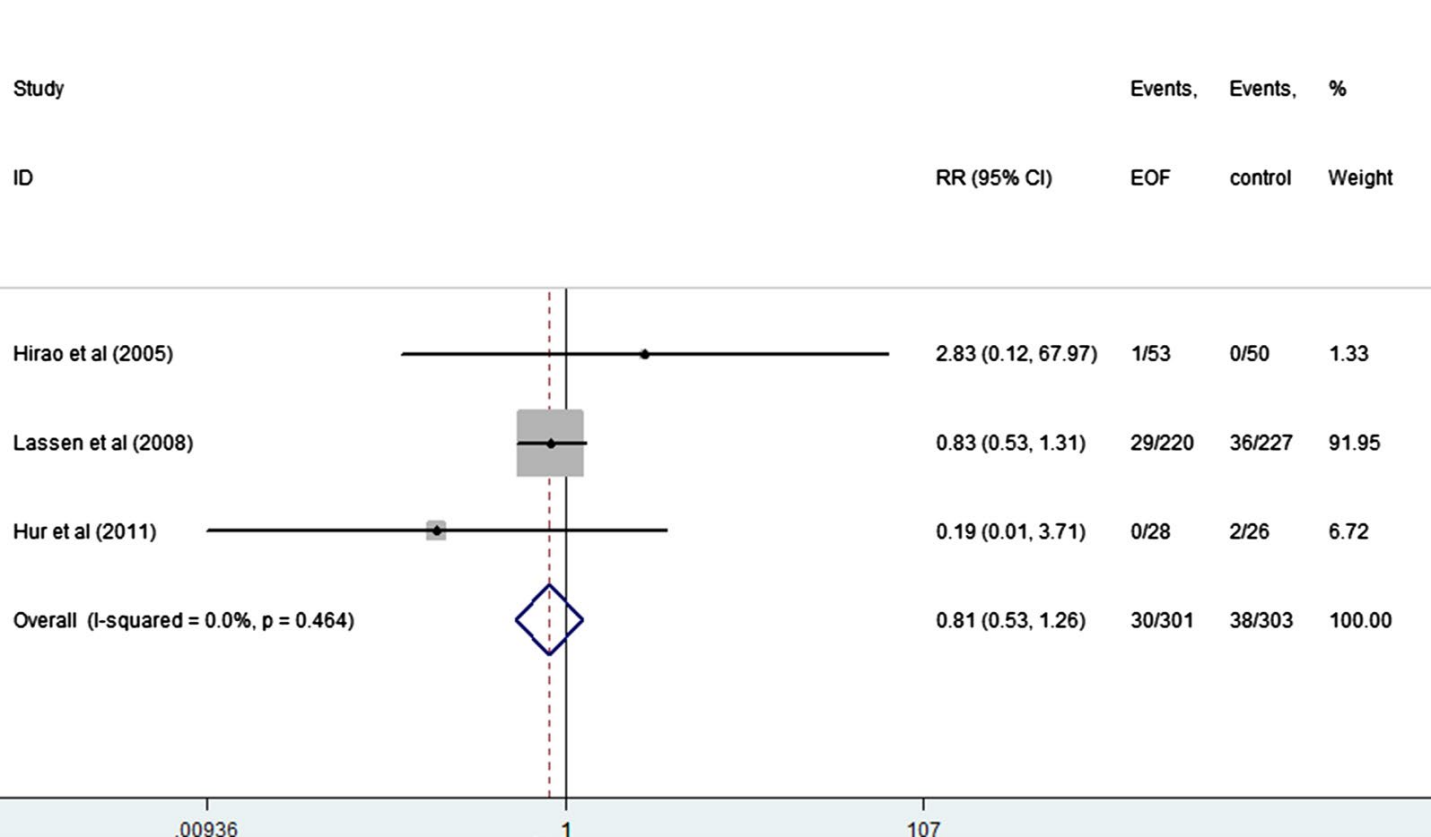
Forest plot of the accidence of reoperation in EOF group and the DOF group. *RR* risk ratio, *EOF* early oral feeding, *DOF* delay oral feeding

**Fig. 10:**
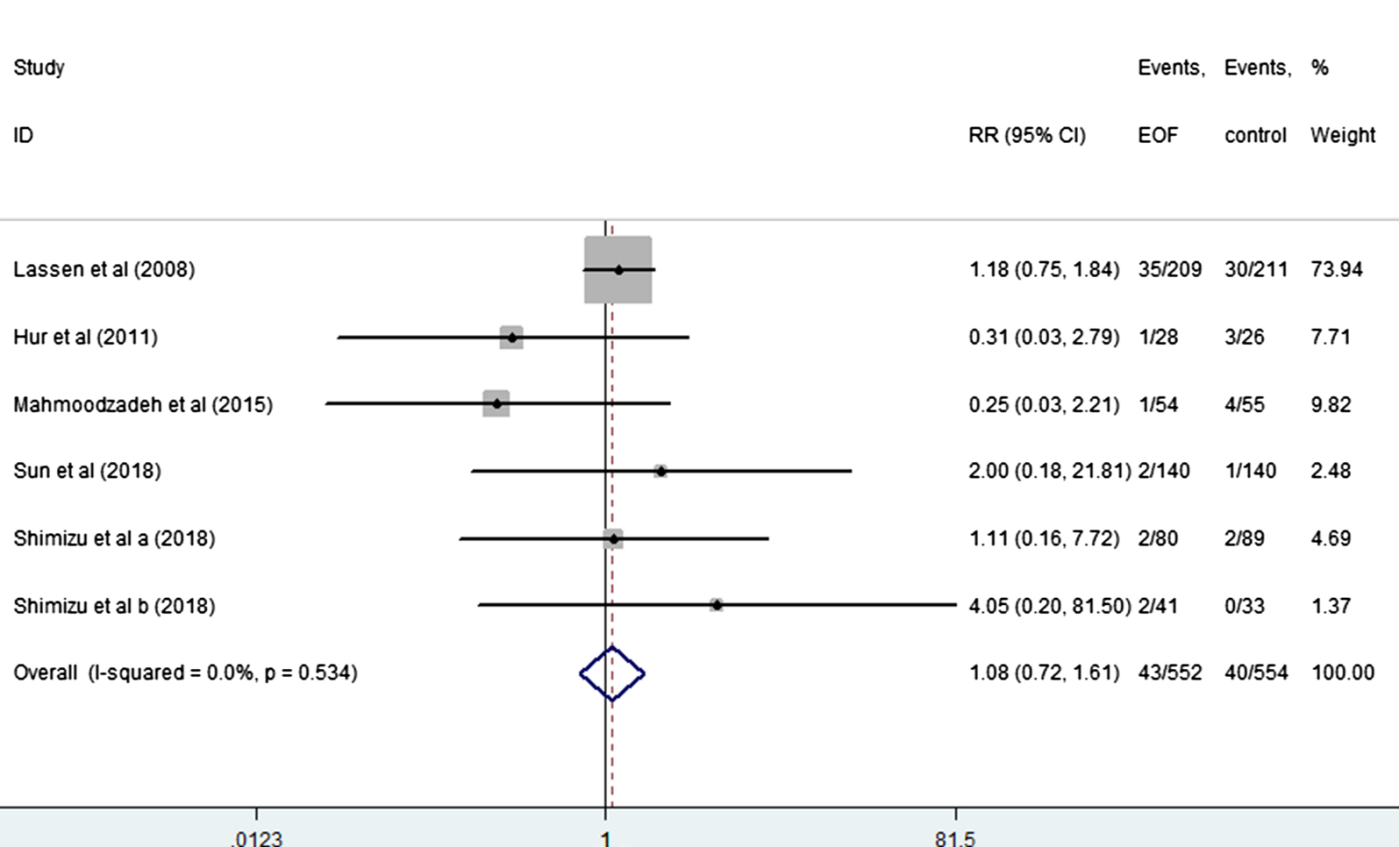
Forest plot of the accidence of readmission in EOF group and the DOF group. *RR* risk ratio, *EOF* early oral feeding, *DOF* delay oral feeding

**Fig. 11:**
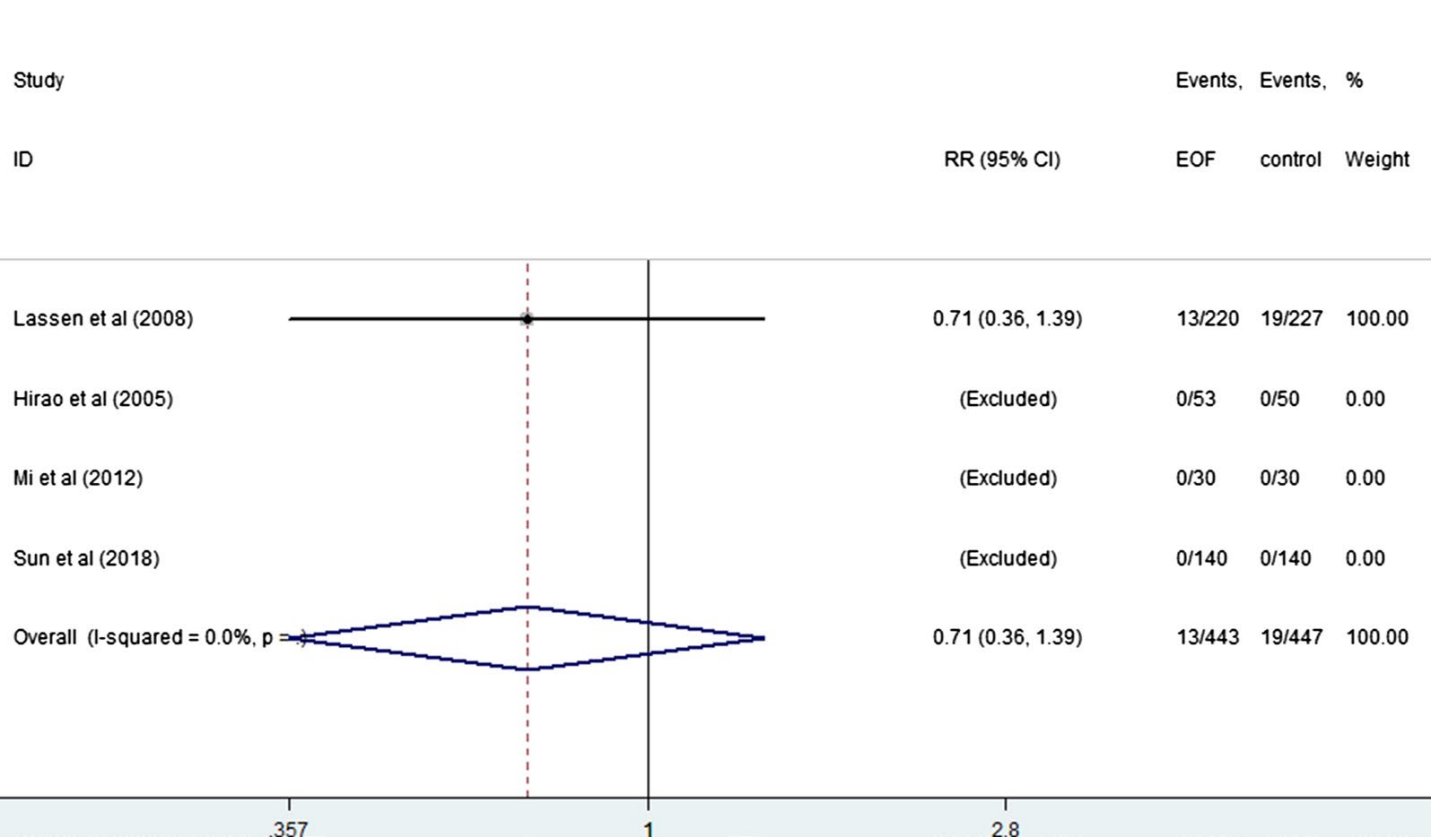
Forest plot of the mortality in EOF group and the DOF group. *RR* risk ratio, *EOF* early oral feeding, *DOF* delay oral feeding

EOF could reduce the risk of pneumonia compared with delay oral feeding (8.4% vs 11.5%) (RR: 0.74, 95% CI 0.55 to 0.99, I^2^ = 0.0%) (Fig. 5). And there was no significant difference between EOF group and delay oral feeding group in the risk of anastomotic leak (RR: 0.91, 95% CI 0.60 to 1.38, I^2^ = 0.0%), anastomotic bleeding (RR: 1.47, 95% CI 0.53 to 4.03, I^2^ = 0.0%), abdominal abscess(RR: 0.54, 95% CI 0.27 to 1.07, I^2^ = 0.0%), reoperation (RR: 0.81, 95% CI 0.53 to 1.26, I^2^ = 0.0%), readmission (RR: 1.08, 95% CI 0.72 to 1.61, I^2^ = 0.0%) and mortality(RR: 0.71, 95% CI 0.36 to 1.39, I^2^ = 0.0%) as shown in Figs. 6, 7, 8, 9, 10, 11.

### Sensitivity analysis and publication bias

**Fig. 12:**
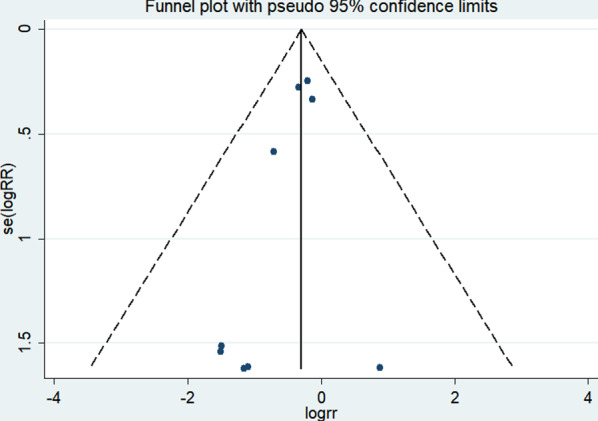
Funnel plot of pneumonia in EOF group and DOF group. *RR* risk ratio, *EOF* early oral feeding, *DOF* delay oral feeding

**Fig. 13:**
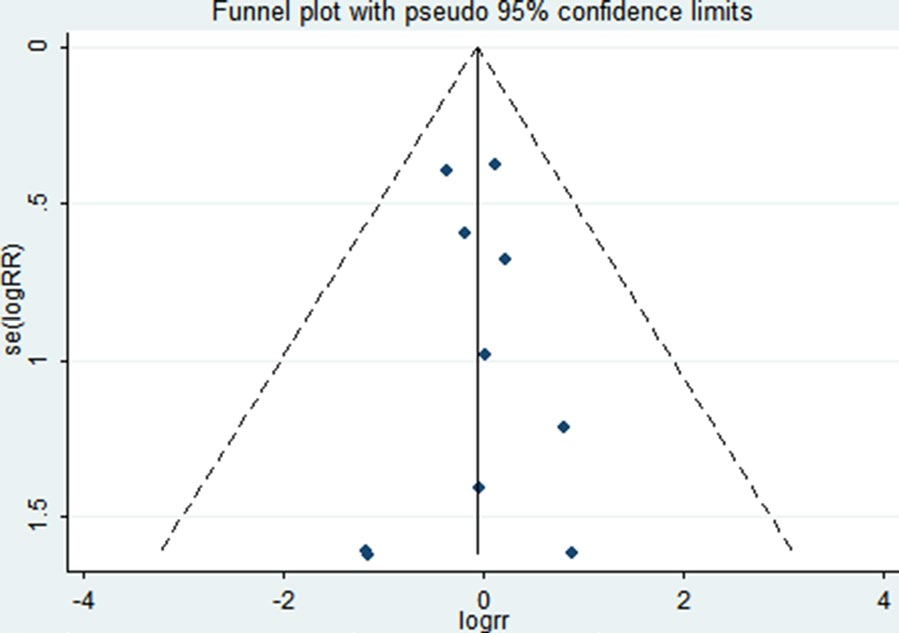
Funnel plot of anastomotic leak in EOF group and DOF group. *RR* risk ratio, *EOF* early oral feeding, *DOF* delay oral feeding

**Fig. 14:**
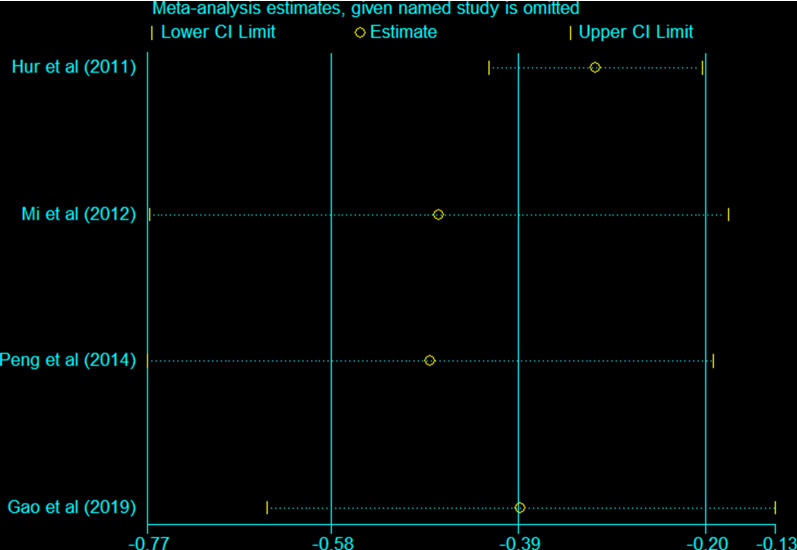
Sensitivity analysis of time of first exhaust in the EOF group and DOF group. *RR* risk ratio, *EOF* early oral feeding, *DOF* delay oral feeding

The funnel plot shows a low probability of publication bias for the included studies, as shown in Figs. 12, 13. The results of the sensitivity analysis are shown in Fig. 14.

## Discussion

ERAS promotes global evidence-based treatment in perioperative period [29]. It was first described and performed in patients after elective colorectal surgery in European countries [30], and now it gradually extends to any type of surgery, including some major upper gastrointestinal surgery [31]. The program aims to reduce injury caused by surgery, support the recovery of intestinal function and promote early activity of patients [32]. It has adopted a variety of strategies in the postoperative process, such as avoiding nasotracheal intubation, epidural analgesia, early exercise and early oral nutrition, most of which show obvious effects. Among them, early oral feeding is different from the traditional oral feeding. EOF program recommends to begin to oral fluid food within postoperative day 1, and gradually transition to semi-fluid and solid diet [33]. The traditional treatment is “nil by mouth” until intestinal function recovers naturally. It is due to fear of postoperative complications, such as anastomotic fistula and aspiration pneumonia [34].

Nowadays, there are only a few meta-analyses to study the efficacy and safety of EOF in patients after upper gastrointestinal surgery. Zhang et al. [12] assessed the effect of EOF on the incidence of anastomotic leakage after esophagectomy through a meta-analysis. And they found that EOF did not increase anastomotic leakage rate. However, due to significant heterogeneity, bias and small samples, the results are unreliable. Another study by Liu et al. [11] made a meta-analysis based on RCTs to evaluate the feasibility of EOF after gastrectomy for gastric cancer. And they suggested that EOF after gastric cancer surgery seems to be feasible and safe regardless of the scope and type of gastrectomy. However, only patients from China and Korea were included, which is not representative of a broad population. Li et al. [35] evaluate the effect of EOF on anastomotic leakage rate after esophagectomy. They concluded that anastomotic leakage in open esophagectomy is related to the timing of oral feeding, and delayed oral feeding is beneficial to reduce anastomotic leakage. However, there was no significant difference in anastomotic leakage between EOF and delayed oral feeding in patients with minimally invasive esophagectomy. This conclusion is contrary to the most meta-analyses. It may be that most of the included studies in the meta-analysis are retrospective studies with low quality, which may lead to low credibility of the conclusion. Willcutts et al. [13] also conducted a meta-analysis to compare the effect of EOF on clinical outcome after upper gastrointestinal surgery. They concluded that compared with conventional feeding, postoperative EOF was not associated with an increase in clinically relevant complications and was associated with a shorter length of hospital stay. There was inherent clinical heterogeneity in their meta-analysis because studies on multiple types of upper gastrointestinal surgery (gastrectomy, esophagectomy, hepatobiliary, and others) were pooled together.

Our meta-analysis evaluated the efficacy and safety of EOF in patients after upper gastrointestinal surgery. The results showed that compared with the traditional oral feeding group, EOF after upper gastrointestinal surgery significantly shorten the LOS and time of first exhaust. EOF also reduced the risk of pneumonia (RR: 0.74, 95% CI 0.55 to 0.99, I^2^ = 0.0%). And there is no significant difference in the risk of anastomotic leak, anastomotic bleeding, abdominal abscess, reoperation, readmission and mortality.

There is a large heterogeneity in the endpoint of the time of first exhaust (I^2^ = 62.1%). Through sensitivity analysis, we found that the heterogeneity mainly comes from the study of Hur et al. [20] We consider that this may be because the sample size of Hur’s study is small (n = 54). And the average age of the Hur’s study is unknown. We know that there are great differences in the tolerance of patients of different ages to surgery. Young people have significantly better tolerance than the elderly. Besides, these studies are conducted in different countries that the standard surgical practice may varies. Hur’s study [20] is from Korea and the other three studies are from China, which may lead to methodological heterogeneity.

The potential clinical implications of this meta-analysis are as follows: (1) this is an updated meta-analysis to evaluate the efficacy and safety of EOF in patients after upper gastrointestinal surgery. Compared to previous studies, we included 12 RCTs that contained a large sample size of 1771 participants; (2) Sensitivity analyses and subgroup analyses were conducted to decompose heterogeneity and explore the influence of sample size on the overall effect; (3) All the included studies were RCTs and the literature was of high quality; (4) Compared with previous studies, literature from different regions was included in this study, such as China, Japan, India, Norway, Iran, Netherlands, Sweden and USA, which was widely representative; (5) The heterogeneity of this meta-analysis is low and the conclusions are more reliable; (6) Only 2 of the studies [18, 28] had sample sizes less than 50; and (7) EOF not only did not increase the risk of pneumonia, but can significantly reduce the risk of pneumonia, which is different from the conclusion of previous studies. And it might be another potential benefit of EOF in upper gastrointestinal surgery, which needs to be further confirmed by higher quality RCTs.

The limitations of our study are as follows: (1) the types of surgery were mostly esophagectomy and gastrectomy. Other types of surgery accounted for a lower proportion; (2) Most of the EOF groups in the RCTs started the oral feeding within POD 1, but there was a large variation in when the control group started oral feeding. It leads to methodological heterogeneity; (3) Several baseline characteristics (diabetes, coronary heart disease, hypertension and neoplasm staging) were not considered which may lead to mixed bias; (4) Most of included RCTs didn’t describe the blinding method used, which may lead to confounding bias; and (5) The endpoint of LOS in half of the included studies had only the mean but no standard deviation, which made it impossible to use these data to calculate the effect size.

In summary, our meta-analysis has demonstrated that compared with the traditional oral feeding group, EOF could significantly shorten the LOS and time of first exhaust after upper gastrointestinal surgery. EOF also reduced the risk of pneumonia. There is no significant difference in the risk of other complications.

Future postoperative strategies for EOF in upper gastrointestinal surgery require safer and more effective multidisciplinary collaboration under better uniform standards and extraction of more large, high-quality samples for evidence-based analysis.

## Data Availability

Please contact author for data requests.

## Abbreviations

EOF: Early oral feeding
DOF: Delay oral feeding
ERAS: Enhanced recovery after surgery
LOS: Length of stay
RCTs: Randomized controlled trials
WMD: Weighted mean difference
RR: Risk ratios
CI: Confidence intervals

## References

[1] Ishida Y, Inaba K, Suda K (2015). Upper gastrointestinal surgery on the esophagus and stomach. Nihon Geka Gakkai Zasshi..

[2] Choi HJ, Lee BI, Kim JJ (2013). The temporary placement of covered self-expandable metal stents to seal various gastrointestinal leaks after surgery. Gut Liver.

[3] Zevallos VF, Herencia LI, Chang F (2014). Gastrointestinal effects of eating quinoa (*Chenopodium quinoa* Willd.) in celiac patients. Am J Gastroenterol.

[4] Dressman JB, Bass P, Ritschel WA (2010). Gastrointestinal parameters that influence oral medications. J Pharm Sci.

[5] Ye RC, Yun JH, Choi SH (2020). Effect of early enteral nutrition on the incidence of acute acalculous cholecystitis among trauma patients. Asia Pac J Clin Nutr.

[6] Alverdy J, Gilbert J, Defazio JR (2014). Proceedings of the 2013 A.S.P.E.N. research workshop: the interface between nutrition and the gut microbiome: implications and applications for human health. JPEN J Parenter Enteral Nutr.

[7] Melloul E, Hübner M, Scott M (2016). Guidelines for perioperative care for liver surgery: enhanced recovery after surgery (ERAS) society recommendations. World J Surg.

[8] Gianotti L, Nespoli L, Torselli L (2011). Safety, feasibility, and tolerance of early oral feeding after colorectal resection outside an enhanced recovery after surgery (ERAS) program. Int J Colorectal Dis.

[9] Lassen K, Kjaeve J, Fetveit T (2008). Allowing normal food at will after major upper gastrointestinal surgery does not increase morbidity: a randomized multicenter trial. Ann Surg.

[10] Le Guen M, Fessler J, Fischler M (2014). Early oral feeding after emergency abdominal operations. Curr Opin Clin Nutr Metab Care.

[11] Liu X, Wang D, Zheng L (2014). Is early oral feeding after gastric cancer surgery feasible? A systematic review and meta-analysis of randomized controlled trials. PLoS ONE.

[12] Zhang C, Zhang M, Gong L (2020). The effect of early oral feeding after esophagectomy on the incidence of anastomotic leakage: an updated review. Postgrad Med.

[13] Willcutts KF, Chung MC, Erenberg CL (2016). Early oral feeding as compared with traditional timing of oral feeding after upper gastrointestinal surgery: a systematic review and meta-analysis. Ann Surg.

[14] Stewart LA, Clarke M, Rovers M (2015). Preferred reporting items for a systematic review and meta-analysis of individual participant data: the PRISMA-IPD statement. J Am Med Assoc.

[15] Sterne J, Savovi J, Page MJ (2019). RoB 2: a revised tool for assessing risk of bias in randomised trials. BMJ Clin Res.

[16] Jiang M, Liu S, Deng H (2021). The efficacy and safety of fast track surgery (FTS) in patients after hip fracture surgery: a meta-analysis. J Orthop Surg Res..

[17] Jiang M, Deng H, Chen X (2020). The efficacy and safety of selective COX-2 inhibitors for postoperative pain management in patients after total knee/hip arthroplasty: a meta-analysis. J Orthop Surg Res..

[18] Suresh V (2000). Feasibility and safety of early oral feeding after cervical esophagogastrostomy. Internet J Thorac Cardiovasc Surg.

[19] Hirao M, Tsujinaka T, Takeno A (2005). Patient-controlled dietary schedule improves clinical outcome after gastrectomy for gastric cancer. World J Surg.

[20] Hur H, Kim SG, Shim JH (2011). Effect of early oral feeding after gastric cancer surgery: a result of randomized clinical trial. Surgery.

[21] Mi L, Zhong B, Zhang DL (2012). Effect of early oral enteral nutrition on clinical outcomes after gastric cancer surgery. Zhong hua Wei chang Wai ke Za zhi.

[22] Peng S-S (2014). Clinical effects of early postoperative oral feeding versus traditional oral feeding after bilioenteric anastomosis. World Chin J Digestol..

[23] Mahmoodzadeh H, Shoar S, Sirati F (2015). Early initiation of oral feeding following upper gastrointestinal tumor surgery: a randomized controlled trial. Surg Today.

[24] Sun HB, Li Y, Liu XB (2018). Early oral feeding following mckeown minimally invasive esophagectomy: an open-label, randomized, controlled noninferiority trial. Ann Surg.

[25] Shimizu N, Oki E, Tanizawa Y (2018). Effect of early oral feeding on length of hospital stay following gastrectomy for gastric cancer: a Japanese multicenter, randomized controlled trial. Surg Today.

[26] Gao L, Zhao ZG, Zhang LY (2019). Effect of early oral feeding on gastrointestinal function recovery in postoperative gastric cancer patients: a prospective study. J BUON.

[27] Berkelmans GHK, Fransen LFC, Dolmans-Zwartjes ACP (2020). Direct oral feeding following minimally invasive esophagectomy (NUTRIENT II trial): an international, multicenter open-label randomized controlled trial. Ann Surg.

[28] Masood A, Viqar S, Zia N (2021). Early oral feeding compared with traditional postoperative care in patients undergoing emergency abdominal surgery for perforated duodenal ulcer. Cureus.

[29] Jiang X, Zhang XF (2019). Anesthesia management on enhanced recovery after surgery (ERAS) during perioperative period in primary hospitals. World Latest Med Inform..

[30] Pisarska M, Pędziwiatr M, Małczak P (2016). Do we really need the full compliance with ERAS protocol in laparoscopic colorectal surgery? A prospective cohort study. Int J Surg..

[31] Pdziwiatr M, Mavrikis J, Witowski J (2018). Current status of enhanced recovery after surgery (ERAS) protocol in gastrointestinal surgery. Med Oncol.

[32] Han-Geurts I, Hop W, Kok N (2010). Randomized clinical trial of the impact of early enteral feeding on postoperative ileus and recovery. Br J Surg.

[33] Muller S, Zalunardo MP, Hubner M (2009). A fast-track program reduces complications and length of hospital stay after open colonic surgery. Rev Gastroenterol Mex.

[34] Nimmo W (2015). Pharmacology of "nil by mouth" after surgery. BMJ.

[35] Li X, Yan S, Ma Y (2020). Impact of early oral feeding on anastomotic leakage rate after esophagectomy: a systematic review and meta-analysis. World J Surg.

